# EXIF Custom: Automatic image metadata extraction for Scratchpads and Drupal

**DOI:** 10.3897/BDJ.1.e973

**Published:** 2013-09-16

**Authors:** Ed Baker

**Affiliations:** † The Natural History Museum, London, United Kingdom

**Keywords:** Scratchpads, image metadata, Drupal, EXIF, XMP, IPTC

## Abstract

Many institutions and individuals use embedded metadata to aid in the management of their image collections. Many deskop image management solutions such as Adobe Bridge and online tools such as Flickr also make use of embedded metadata to describe, categorise and license images. Until now Scratchpads (a data management system and virtual research environment for biodiversity) have not made use of these metadata, and users have had to manually re-enter this information if they have wanted to display it on their Scratchpad site. The Drupal described here allows users to map metadata embedded in their images to the associated field in the Scratchpads image form using one or more customised mappings. The module works seamlessly with the bulk image uploader used on Scratchpads and it is therefore possible to upload hundreds of images easily with automatic metadata (EXIF, XMP and IPTC) extraction and mapping.

## Introduction

The use of embedded image metadata is becoming widespread in the biodiversity informatics community (e.g. Stafford et al. 2010 & Tulig et al. 2012), and is frequently used to describe the subject and licencing of images as well as for recording the 'tombstone metadata' (e.g. Introduction to Metadata) - when the image was created, last edited, who created it, and where and how it was created.

The eMonocot project (http://about.e-monocot.org) makes use of the Scratchpads (Smith et al. 2011) infrastructure as a tool for collecting, curating, and creating content to be harvested by the eMonocot portal (http://e-monocot.org). As part of this project hundreds of images with embedded metadata are being uploaded to a number of different Scratchpads, combined with images directly uploaded by partner communities, and exported en mass to the portal. For this to be technically feasible at scale images from varied, disparate sources need to have their metadata standardised as part of the bulk upload process.

There are three widespread image metadata formats that can be handled by this module. A subset of the EXIF standard (Camera and Imaging Products Association Standardization Committee 2010) specifies a method for tagging of images with metadata. This is widely used by device manufacturers to record both the make and model of the image capture device and also the device's settings when the image was captured (e.g. focal length, flash duration). The eXtensible Metadata Platform (XMP) was originally developed by Adobe Systems Incorporated and later adopted by the International Standards Organisation as ISO 16684-1:2012. It uses a data model defined in Adobe 2012 which is serialised in XML when embedded into files. The International Press Telecommunications Council defines the IPTC Core and Extension metadata standards (IPTC 2010).

An existing Drupal module, Exif (https://drupal.org/project/exif), provides a mechanism for displaying embedded image metadata on Drupal nodes, but does not provide a mechanism for mapping the metadata into fields. The import of embedded metadata into Scratchpads/Drupal fields is a requirement of the eMonocot project and is useful for the wider Scratchpads community as it allows for these data to be easily used by other Drupal modules (e.g. Views - https://drupal.org/project/views) and in other Scratchpads-specific functions such as our on-going work on implementing the ability to export data via DarwinCore Archives (GBIF DarwinCore Archives). There is a comparison of these two modules (and potentially other similar Drupal modules) at https://drupal.org/node/1842686.

## Web location (URIs)

Homepage: https://drupal.org/project/exif_custom

Download page: https://drupal.org/node/1826526/release

Bug database: https://drupal.org/project/issues/exif_custom

## Technical specification

Platform: Scratchpads/Drupal

Programming language: PHP

Interface language: English

## Repository

Type: Git

Browse URI: http://drupalcode.org/project/exif_custom.git

## Usage rights

### Use license

Other

### IP rights notes

The source code of this module is hosted on https://drupal.org. All content on the Drupal.org itself is copyrighted by its original contributors, and is licensed under the Creative Commons Attribution-ShareAlike license 2.0 and is also available under the GPL version 2 or later.

## Implementation

### Implements specification

**Figure 1.**
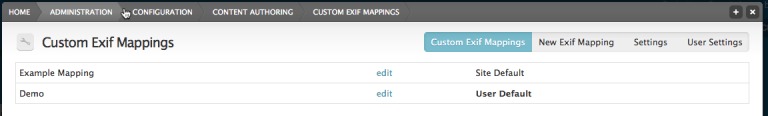
Multiple image mappings, showing which are set at the Site Default and User Default - and which will be used for the currently logged-in user in bold.

EXIF, XMP and IPTC are the three image metadata standards in widespread use and the eMonocot project (http://e-monocot.org) (which uses Scratchpads) makes use of all three systems. Due to the flexibility of these systems, particularly of XMP, it is possible that the same field can be defined in more than one of these standards and there is no guarantee that all Scratchpads users will use the same image metadata field for the same data. Scratchpads as a system (and also Drupal on which Scratchpads are built) are highly customisable, and users may create their own custom fields to add metadata to image files. Due to this multiplicity of possible input formats it is not desirable for the module described here to define a mapping of embedded image metadata fields to Scratchpads/Drupal fields (adding fields to images in Drupal requires the File Entity module - https://drupal.org/project/file_entity). Users may also want to upload images from a number of different sources that make use of different subsets of the three image metadata standards supported. For these reasons this module allows users to define any number of named mappings between embedded image metadata and the Scratchpads/Drupal image fields. It is possible for those with the necessary privileges on the system to define the default mapping used by the site, and for individual users to override this with their own choice of User Default mapping (Fig. 1).

**Figure 2.**
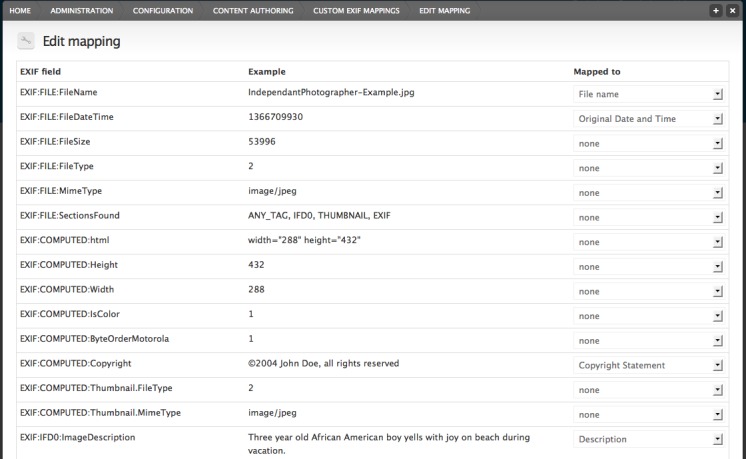
Mapping embedded image metadata fields to Scratchpads/Drupal image fields. The image uploaded is a standard testing image from the EXIF Toolkit available from http://iptc.org. The Scratchpads project recommends the use of Creative Commons open licences.

The configuration pages for this module can be found under 'Custom Exif Mappings' in the standard Scratchpads/Drupal administration interface. The 'Settings' tab allows those with the required privileges to set the site's default mapping, and to turn on or off the automatic saving of embedded image metadata to the image fields of the site when an image is uploaded. The 'User Settings' tab provides an interface for individual users to override the site's default mapping with a mapping of their choice. New mappings can be created through the 'New Exif Mapping' tab. The first step in this process is to name the new mapping and upload a sample image that contains all of the metadata fields that you want to map to Drupal/Scratchpads image fields. The module extracts all of the embedded metadata fields that have values assigned, and provides a form that displays the name of the embedded metadata field, an example value from the sample image, and a drop-down list of Scratchpads/Drupal fields that can be mapped to (Fig. 2).

Once one or more mappings have been created, and the Site Default and/or User Default mapping has been set, the Scratchpads/Drupal fields will automatically be populated from the embedded image metadata when a new file entity is created - either through individual entity creation or through batch entity creation (using a module such as Plupload - https://drupal.org/project/plupload) to upload multiple images at once.

### Audience

This module can be enabled on Scratchpads sites via the Tools page in Admin > Structure. It can also be downloaded by maintainers of other Drupal sites from Drupal.org and enabled in the same way as any other module.

The module is potentially useful for anybody who wants to extract embedded metadata from uploaded images and use it in fields on a Drupal site. By making metadata available in fields, the metadata can be exposed to other third-party modules such as Views (https://drupal.org/project/views), allowing for many display options and filtering opportunities.

The eMonocot content team based at the Royal Botanic Gardens Kew make use of this module on the eMonocot Scratchpads to bulk upload images which have had their metadata curated using Adobe Bridge. The module extracts the metadata embedded within the image into Drupal fields which allows for both display of this data on the Scratchpad and also in the DarwinCore Archive file that is used to contribute Scratchpad data to the eMonocot portal and also the Encyclopedia of Life. This workflow prevents images being separated from their accompanying metadata (through metadata embedding) and also saves time and effort - previously in Drupal performing the task of importing metadata would have required either copying and pasting data or spreadsheet manipulation using a number of different import tools.

## Additional information

### Dependencies

This module requires the Drupal File Entity (https://drupal.org/project/file_entity) module.

### Integration

The module will work with any fields associated with image file entities in Drupal. The Scratchpads Audubon Core module (https://git.scratchpads.eu/v/scratchpads-2.0.git/tree/HEAD:/sites/all/modules/custom/scratchpads/scratchpads_audubon_core) creates a set of fields that are compliant with the current version of Audubon Core (a standard metadata schema for images of biological specimens and observations). With the EXIF Custom module it is possible to map embedded image metadata directly to these fields when the images are uploaded. The use of standard Drupal fields means that it is also possible to expose embedded metadata to external services, notably via DarwinCore Archives using the Scratchpads DarwinCore Archive Export module (https://git.scratchpads.eu/v/scratchpads-2.0.git/tree/HEAD:/sites/all/modules/custom/dwca_export).
